# Repeatability of quantitative parameters of 18F-fluoride PET/CT and biochemical tumour and specific bone remodelling markers in prostate cancer bone metastases

**DOI:** 10.1186/s13550-017-0289-9

**Published:** 2017-05-15

**Authors:** Cecilia Wassberg, Mark Lubberink, Jens Sörensen, Silvia Johansson

**Affiliations:** 1 Department of Surgical Sciences, Section of Nuclear Medicine and PET, Uppsala, Sweden; 2 Department of Immunology, Genetics and Pathology, Section of Oncology, Uppsala University, Uppsala, Sweden; 3 PET Centre, Uppsala University Hospital, Entrance 79, 5th floor, S-75185 Uppsala, Sweden

**Keywords:** 18F-fluoride PET, Bone metastases, Prostate cancer, Test-retest, Repeatability, Bone markers, Translational

## Abstract

**Purpose:**

18F-fluoride PET/CT exhibits high sensitivity to delineate and measure the extent of bone metastatic disease in patients with prostate cancer. 18F-fluoride PET/CT could potentially replace traditional bone scintigraphy in clinical routine and trials. However, more studies are needed to assess repeatability and biological uptake variation. The aim of this study was to perform test-retest analysis of quantitative PET-derived parameters and blood/serum bone turnover markers at the same time point.

Ten patients with prostate cancer and verified bone metastases were prospectively included. All underwent two serial 18F-fluoride PET/CT at 1 h post-injection. Up to five dominant index lesions and whole-body 18F-fluoride skeletal tumour burden were recorded per patient. Lesion-based PET parameters were SUVmax, SUVmean and functional tumour volume applying a VOI with 50% threshold (FTV_50%_). The total skeletal tumour burden, total lesion 18F-fluoride (TLF), was calculated using a threshold of SUV of ≥15. Blood/serum biochemical bone turnover markers obtained at the time of each PET were PSA, ALP, S-osteocalcin, S-beta-CTx, 1CTP and BAP.

**Results:**

A total of 47 index lesions and a range of 2–122 bone metastases per patient were evaluated. Median time between 18F-fluoride PET/CT was 7 days (range 6–8 days). Repeatability coefficients were for SUVmax 26%, SUVmean 24%, FTV_50%_ for index lesions 23% and total skeletal tumour burden (TLF) 35%. Biochemical bone marker repeatability coefficients were for PSA 19%, ALP 23%, S-osteocalcin 18%, S-beta-CTx 22%, 1CTP 18% and BAP 23%.

**Conclusions:**

Quantitative 18F-fluoride uptake and simultaneous biochemical bone markers measurements are reproducible for prostate cancer metastases and show similar magnitude in test-retest variation.

## Background

The clinical gold standard for detecting and defining disease extent of skeletal metastasis has been conventional ^99m^Tc-methylene diphosphonate planar bone scintigraphy (BS) or SPECT with or without computed tomography (CT). BS has moderate sensitivity for osteoblastic bone lesions such as in prostate and breast cancer, but the method shows low specificity due to skeletal uptake in degenerative changes and other benign conditions such as trauma and infection. An alternative to BS in prostate cancer is PET/CT with 18F-sodium fluoride (18F-fluoride PET). 18F-fluoride PET offers many advantageous technical features with superior pharmacokinetics and image quality, better tumour-to-background ratio due to faster blood clearance and higher resolution. Previous studies have shown that 18F-fluoride PET/CT has better accuracy and detects more bone metastases and at an earlier time compared to BS in prostate cancer patients [1–4]. Many centres with access to increasing number of PET/CT scanners and radiotracers discuss the replacement of BS with 18F-fluoride PET/CT technique. BS is an approved imaging biomarker of disease progression, and serial imaging is integrated into numerous clinical trials. One important aspect regarding clinical adoption of 18F-fluoride PET concerns to what extent the PET method can substitute BS as a biomarker in clinical trials.

One advantage of PET over conventional BS is that 18F-fluoride SUV correlates well with kinetic parameters of bone formation [5]. This allows whole-body PET to be used for quantitative studies, which might be useful for defining prediction and determination of therapy response and prognosis using imaging at early time points. Baseline and follow-up imaging in clinical studies quantifying lesional uptake such as SUVmax, SUVmean and functional tumour volume (FTV); total skeletal tumour burden; total lesion 18F-fluoride uptake (TLF) and biochemical bone turn over markers might correlate with outcome. In castrate resistant prostate cancer, skeletal tumour burden including biochemical bone parameters is suggested to be strong prognostic indicators of overall survival [6]. Thus, 18F-fluoride can be a potential biomarker for monitoring treatment response and outcome.

A reproducible method and robust imaging protocol is crucial for serial imaging at baseline and during treatment in studies, and for these reasons, awareness of the biological variation in qualitative and quantitative 18F-fluoride uptake and tracer distribution is of relevance when interpreting data. The test-retest repeatability of quantitative aspects of fluoride PET under standardised conditions is not known.

Both BS and fluoride PET are indirect markers of bone metastatic growth, and signal is directly proportional to the formation of new bone. The biological variation in bone metastases of prostate cancer is also repeatedly measured with biochemical markers of tumour activity derived from blood sampling, especially PSA doubling time and ALP (alkaline phosphatase), and more specific bone remodelling markers such as S-osteocalcin (bone protein: bone γ-carboxylglutamic acid-containing protein), S-beta-CTx (degraded fragments of collagen fibrils including C-terminal telopeptide (CTx)), 1CTP (pyridinoline cross-linked carboxy-terminal telopeptide of type I collagen) and BAP (bone-specific alkaline phosphatase) [7–10]. The covariation of these blood-based biomarkers towards quantitative fluoride PET has not been studied.

The aim of this study was to investigate the repeatability of quantitative 18F-fluoride PET-derived parameters using both selected index lesions and total skeletal tumour burden in two serial PET/CT examinations and correlate biochemical bone remodelling markers at the same time point.

## Methods

### Patients

This was a prospective study in patients with biopsy-verified prostate cancer with bone metastases. Patients were recruited from the Oncology Department at Uppsala University Hospital between August 2013 and February 2014. All patients gave written informed consent. A total of ten patients were included and underwent two serial 18F-fluoride PET and blood sampling with biochemical bone remodelling markers twice seven days apart. Blood sampling was performed in the fasting state in the morning directly followed by PET. All patients were on stable treatment regimens for at least four weeks prior to study start. Change in medical treatment between the first and second study date was an exclusion criteria.

### Imaging protocol

The imaging technique was performed according to a recommended clinical protocol [11]. The patients were well hydrated and requested to empty their bladder directly before scanning. Examination was performed after intravenous administration of 3 MBq/kg of 18F-labeled sodium fluoride at 1 h after injection.

Examinations were performed on a Discovery ST PET/CT Scanner (GE Healthcare, Waukesha, WI): field of view (FOV) of 15.7 cm in axial and 70 cm in transaxial direction and slice thickness of 3.27 mm. Patients were positioned supine with arms elevated. An unenhanced CT (40–80 mA) from mid-thigh to vertex of the skull was used for attenuation correction. Images were acquired in 3D mode with an acquisition time of 3 min/bed position. The images were reconstructed iteratively with ordered subset expectation maximisation (2 iterations, 21 subsets, 5 mm Gaussian post-filter), applying all relevant corrections according to the recommendations of the manufacturer, to a 128 × 128 matrix in a 50-cm FOV and fused with unenhanced CT.

### Image analysis

#### Five index lesions

**Fig. 1:**
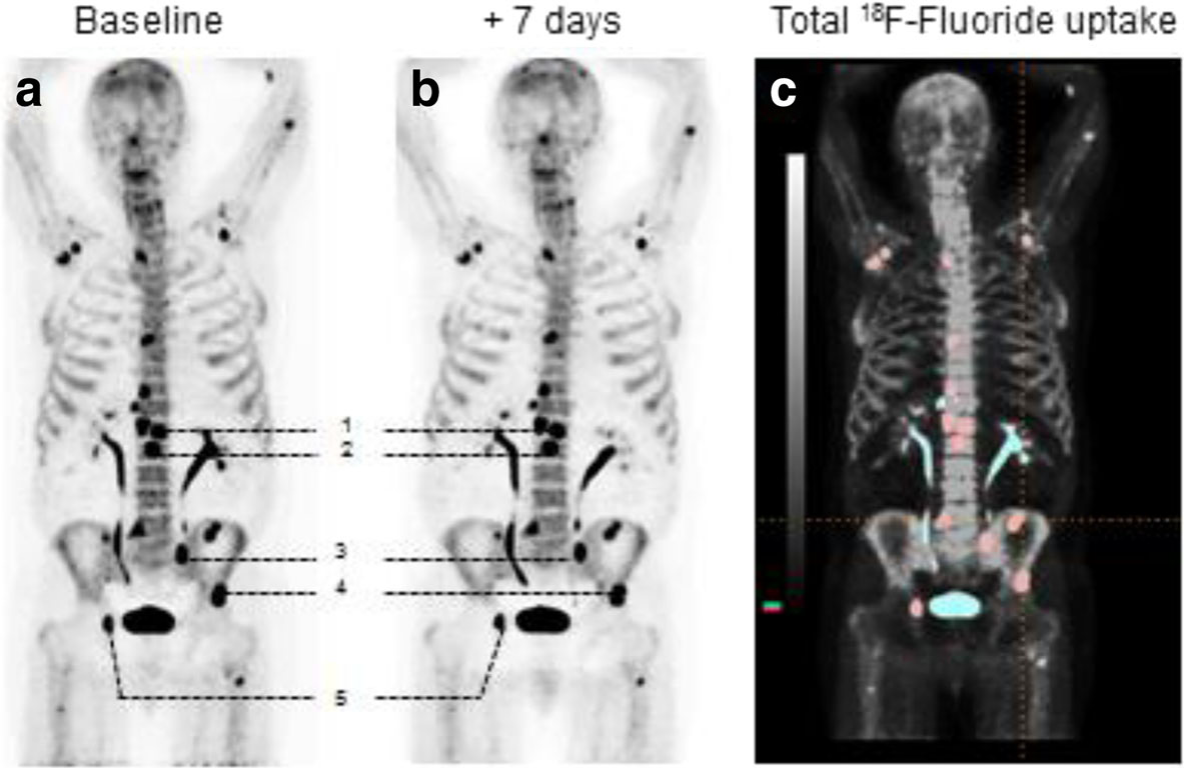
Patient 8 at baseline (**a**) and at +7 days (**b**) performing PET1 and PET2. Five chosen index lesions (numbered 1–5) were delineated for VOI measuring SUVmax, SUVmean, FTV_50%_ and total lesion fluoride uptake at PET/CT (baseline). **c** Included lesions are highlighted in *red* (*n* = 17) and the *blue* uptake represents lesions that were omitted, urine activity and degenerative or equivocal findings

Qualitative and quantitative analyses were performed on a GE Advance Workstation AW v4.4 with semi-automated analysis software PET VCAR. At PET1, up to five dominant index lesions (highest SUVmax) were chosen as representative for testing repeatability (Fig. 1). Only tumours clearly delineated towards physiological uptake and/or other anatomically close bone metastases were included. SUVmax, SUVmean and FTV were measured semi-automatically for each metastasis by applying a VOI with a threshold of 50% of the maximum uptake value delineating each tumour (FTV_50%_). As reference organs of physiological background, uptake of 18F-fluoride tracer was measured in the liver (3-cm-diameter spherical VOI), ascending aorta (1.5-cm-diameter VOI) and muscle (1.5-cm-diameter VOI).

#### Whole-body evaluation

The skeletal tumour burden was evaluated by using a volumetric public domain semi-automated software program, Metavol [12]. A large VOI over the whole-body volume was applied with a threshold of SUV of ≥15 outlining all bone lesions per patient to allow extraction of total 18F-fluoride tumour volume (FTV_SUV15_). The total skeletal tumour burden by 18F-fluoride uptake activity, total lesion 18F-Fluoride burden (TLF), was calculated by summing the product of SUVmean and FTV_SUV15_ for all lesions. The whole-body determination of skeletal tumour burden with 18F-fluoride PET/CT has been reported previously to be feasible and with high inter-reader reproducibility [13]. By correlating all uptakes on PET with CT morphology, we excluded all non-metastatic physiological and degenerative 18F-fluoride uptake. Uptake in a spinal compression fracture was excluded in a single patient.

### Blood sampling

Biochemical tumour and bone remodelling markers, PSA, ALP, S-osteocalcin, S-beta-CTx and BAP were taken and handled by an accredited laboratory at Uppsala University Hospital. The blood samples were taken at the same day as the PET/CT examinations and at the same time point between 8:00 and 10:00 am before breakfast taking the diurnal rhythm of the markers in consideration. 1CTP was analysed by ELISA (UniQ ICTP ELISA 05892, Orion Diagnostica, Espoo, Finland). BAP was analysed (measured with MicroVue BAP kit 8012, Quidel, San Diego, CA, USA). The total coefficient variation (CV) for the assays was 6%.

### Statistical analysis

Correlations were calculated with Pearson correlation coefficients for SUVmax, SUVmean and FTV_50%_ and for whole-body tumour skeletal burden (TLF). The same was performed for biochemical bone turn over markers, PSA, ALP, S-osteocalcin, S-beta-CTx, 1CTP and BAP. Bland-Altman plots were used to evaluate measurement bias [14]. Comparisons of mean values were performed using Student’s *t* test. A two-sided *p* value <0.05 was assumed to be statistically significant. The coefficient of repeatability (CR) for paired measurements was calculated as 2 × SD of the difference between the measurements [15].

## Results

Patient characteristics and on-going treatment are shown in Table 1. Protocol compliance was high and all patients performed PET1 and PET2 with median time between PET1 and PET2 of 7 days (range 6–8 days). Actual injected doses at the two time points were nearly identical (272 ± 39 and 267.6 ± 40 MBq, *P* = not significant, NS). Actual incubation time of the radiotracer was 61.2 ± 1.9 min (range 60–66 min) and for PET2 62.6 ± 2.9 min (range 60–68 min), *P* = NS.

**Table 1:** Patient characteristics and total number of bone metastases with 18F-fluoride PET/CT uptake

Patients’ ID	Age at diagnosis	Gleason at diagnosis	PSA at baseline	Ongoing treatment	Total number of bone metastases
1	82	3 + 4	50	Denosumab, GnRH analogue (goserelin), calcium tablets	27
2	80	4 + 3	0.4	GnRH analogue (goserelin), calcium tablets	15
3	79	4 + 5	14	Anti-androgen (bicalutamid), GnRH analogue (goserelin)	22
4	79	5 + 4	0.5	Denosumab, GnRH analogue (goserelin), dexamethasone, calcium tablets	10
5	77	4 + 5	33	Abiraterone, denosumab, GnRH analogue (goserelin), dexamethasone, calcium tablets	29
6	77	3 + 4	96	Denosumab, GnRH analogue (goserelin),	122
7	71	3 + 4	1000	Denosumab, GnRH analogue (goserelin), prednisolone, calcium tablets	2
8	69	3 + 4	341	Abiraterone, denosumab, GnRH analogue (goserelin), dexamethasone, calcium tablets	17
9	62	5 + 4	43	Denosumab, GnRH analogue (goserelin), calcium tablets	7
10	70	5 + 5	91	Abiraterone, denosumab, prednisolone, calcium tablets	40

**Fig. 2:**
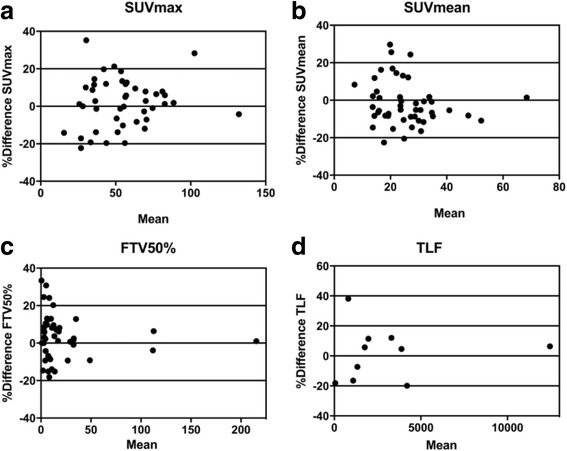
Bland-Altman difference plots in relative values for 18F-fluoride PET repeatability: **a** mean SUVmax, **b** mean SUVmean, **c** mean FTV_50%_ and **d** mean TLF

A total of 47 index lesions with the highest SUVmax (range 2–5 per patient) were chosen and evaluated in ten patients (patient ID 2 only had two lesions in total). They were all osteoblastic and/or mixed lesions with both osteoblastic and lytic CT morphology pattern. The mean size of the index lesions on CT was 3.7 cm (range 1.2–7 cm). For TLF, whole-body skeletal tumour burden, the total number of lesions per patient was in the range of 2–122. Table 2 shows all repeatability data of quantitative PET parameters. Overall correlations were high in all comparisons (*r* > 0.95, *p* < 0.001 for all). Relative CR ranged from 23% for FTV_50%_ to 35% for TLF. Figure 2 graphically shows Bland-Altman plots for PET-derived parameters. No systematic bias was seen, but there was an obvious larger variation for smaller lesions regardless of PET parameter. Uptake in reference organs was reproducible with correlations in the range of *r* = 0.6 to 0.8 with relative CR’s of 18% for mediastinal blood pool to 26% for the liver.

**Table 2:** Repeatability of 18F-fluoride uptake in index lesions and total lesion 18F-fluoride uptake per patient

Patients	Results up to five index lesions per patient						Results of total lesion 18F-fluoride uptake	
	SUVmax (g/mL)		SUVmean (g/mL)		FTV_50%_ (mL)		TLF (g/mL mL)	
	Scan 1	Scan 2	Scan 1	Scan 2	Scan 1	Scan 2	Scan 1	Scan 2
1	47.5	50.5	20.6	21.6	9.3	9.3	1857	2082
2	87.9	83.8	44.1	43.1	3.5	3.5	1381	1284
3	76.2	72.7	31.2	29.3	77.6	79.7	12056	12,854
4	65.5	58.6	31.9	28.6	16.6	17.3	3098	3496
5	45.8	45.0	19.6	19.4	11.9	12.4	1711	1811
6	34.9	32.7	15.3	14.6	27.7	26.6	4614	3780
7	33.6	35.0	14.7	13.4	2.7	3.0	61	51
8	52.0	44.5	26.5	23.0	8.0	9.1	1172	994
9	35.4	42.2	18.5	23.3	7.5	7.3	654	962
10	64.7	64.0	29.6	28.7	28.7	28.3	3790	3970
Mean ± SD	58.4 ± 18.7	58.1 ± 16.6	26.8 ± 9.2	26.4 ± 8.6	20.4 ± 23.0	20.7 ± 22.8	1857 ± 3469	2082 ± 3663
CC	0.95 (95% CI 0.92–0.97) *p* < 0.001		0.97 (95% CI0.95–0.98) *p* < 0.001		1.0 (95% CI 1.0–1.0) *p* < 0.001		0.99 (95% CI 0.98–1.00) *p* < 0.001	
CR absolute	13.6		5.6		3.7		854	
CR relative	26%		24%		23%		35%	

*CC* Pearson correlation coefficient, *CI* confidence interval, *CR* coefficient of repeatability

Table 3 shows repeatability of biochemical bone remodelling markers obtained immediately before PET. Correlations were excellent (*r* > 0.95, *p* < 0.001 for all) and relative CR for all samples was low, ranging from 18% for 1CTP to 23% for ALP. Blood sampling of 1CTP and BAP was not available for patient 2.

**Table 3:** Repeatability of PSA and biochemical bone turnover markers

Patients’ ID	PSA (μg/L)		ALP (μg/L)		S-osteocalcin (μg/L)		S-beta-CTx (ng/mL)		1CTP (ng/mL)		BAP (U/L)	
Scan	1	2	1	2	1	2	1	2	1	2	1	2
1	67	64	1.6	1.3	13	13	96	93	7.0	7.1	21	21
2	0.4	0.4	1.1	1.2	6.5	7.4	248	280	–	–	–	–
3	15	15	1.5	1.6	23	21	784	781	7.4	7.7	22	22
4	0.8	1	0.6	0.56	13	12	87	97	5.8	6.8	7.7	7.2
5	35	37	1.4	1.6	6.2	5.2	101	104	5.0	5.6	32	29
6	106	98	2.9	2.8	32	33	100	104	8.7	8.3	66	72
7	884	799	1.3	1.5	6.6	7.4	106	101	9.2	8.9	14	14
8	157	140	1.3	1.4	17	18	103	101	8.7	7.5	23	22
9	28	27	1.4	1.5	8.3	8.2	32	24	3.6	3.4	22	22
10	120	123	3.5	3.1	7.0	7	43	42	4.5	4.3	56	40
Mean	141 ± 266	130 ± 240	1.7 ± 0.9	1.7 ± 0.7	13.3 ± 8.6	13.2 ± 8.6	170 ± 223	173 ± 224	6.7 ± 2.0	6.6 ± 1.8	29 ± 19	28 ± 19
CC	1.0 (95% CI 1.0–1.0) *p* < 0.001		0.98 (95% CI 0.91–1.0) *p* < 0.001		0.99 (95% CI 0.97–1.0) *p* < 0.001		0.99 (95% CI 0.99–1.0) *p* < 0.001		0.95 (95% CI 0.78–0.99) *p* < 0.001		0.95 (95% CI 0.78–0.99) *p* < 0.001	
CR absolute	53		0.41		2.0		23.0		1.26		11.8	
CR (%)	19%		23%		18%		22%		18%		23%	

*CC* Pearson correlation coefficient, *CI* confidence interval, *CR* coefficient of repeatability

There was no correlation of any PET parameter towards any of the biochemical tumour or bone remodelling markers. ALP correlated linearly with BAP (*r* = 0.89, *p* = 0.001), while no other comparisons reached statistical significance.

## Discussion

This study investigated the repeatability of quantitative 18F-fluoride PET/CT measurements and simultaneously acquired blood-based tumour and bone remodelling markers. Repeatability of five selected index lesions in 18F-fluoride PET/CT was high and appears to be a stable and trustworthy technique that potentially can replace the traditional BS. For evaluating and monitoring therapy effect, a change in SUVmax, SUVmean and FTV_50%_ between the range of 23% up to 26% can occur as a normal variation, suggesting that changes of ≥25% increase or decrease during treatment can be interpreted as a significant change in 18F-fluoride uptake for a single lesion. Similar magnitudes in test-retest variation were observed for PSA and the bone specific remodelling markers in blood/serum. For evaluating and monitoring therapy effects of the total skeletal tumour burden, TLF, changes less than 35% cannot be distinguished from normal test-retest variability. This higher variation in uptake was probably due to inclusion of many small lesions (non-index) that were more prone to variation in uptake SUV and volume than larger lesions in a test-retest setting between PET1 and PET2. The repeatability coefficient used as the statistical estimate of test-retest precision in this study can be considered a conservative measure but is in line with results from studies of FDG-PET [16]. Other frequently used statistics, such as intra-class correlation or mean standard error of measurement, typically indicate much lower variation.

Rohren et al showed that determination of skeletal tumour burden with 18F-fluoride PET/CT is feasible and highly reproducible regarding inter-observer reproducibility [13]. Using a SUVmax threshold of 10, they were able to exclude nearly all non-cancerous bone activity from volumetric calculations. In the current study, a threshold of SUVmax ≥15 was found to perform better for excluding tracer uptake in degenerative changes. The reason for this could be related both to technical and biological issues. 18F-fluoride uptake in bone is irreversible at least for the first few hours after injection and increases with time. Soft tissues function as a reservoir of un-bound tracer from which tracer will recirculate for further uptake in bone or urinary excretion. Excretion is solely through urine. Hence, the actual values derived from regional uptake measurements will vary with the time from tracer injection to scanning, emission acquisition time per bed position and renal clearance. To reduce the impact of these known confounders, patients were instructed to drink water ad libitum prior to and after tracer injection and scanning was performed as close to the 1-h time point as possible in a clinical environment. Sampling for biochemical markers was standardised by obtaining blood at a fixed hour after an over-night fast. By coincidence, PET was therefore also performed in the fasting state, but there is no evidence that fasting affects uptake of 18F-fluoride and is not currently recommended [11].

The translational approach in comparing the repeatability in PSA and specific bone remodelling markers simultaneously with PET/CT might be of value for studies evaluating treatment effects on a systemic level. Microfractures/healing effects can for example explain the biological variation in bone remodelling markers even though no change in treatment has been performed. Some recent studies suggest that 18F-fluoride PET/CT has an impact on treatment monitoring in patients with evidence of progressive osteoblastic bone metastases [17]. The ability to delineate treatment response of systemic therapy in castration-resistant prostate cancer bone metastases with quantitative PET data correlated with bone progression-free survival and alkaline phosphatase (ALP) but not with PSA [18]. The SUVmax, SUVmean, MTV_50%_ or TLF in our study did not correlate directly with PSA or any of the bone remodelling markers. Similar to 18F-fluoride PET, osteocalcin is regarded as a marker of bone formation. Lack of correlation between these suggests that 18F-fluoride PET provides information not available from current blood-based biomarkers.

Traditional bone scintigraphy is still much more widely available than 18F-fluoride PET/CT. Bone scan index (BSI, i.e. total skeletal uptake on ^99m^Tc-diphosphonate bone scanning) has been shown to be a useful predictor of outcome of time to castration-resistant prostate cancer and survival in patients with hormone-sensitive metastatic prostate cancer [19]. The TLF index investigated in this study is similar to BSI, but direct comparisons are needed before 18F-fluoride could be favoured. Both BSI and TLF reflect the osteoblastic reaction to tumour growth and not tumour cell metabolism, as in 18F FDG-PET/CT. Other phenomena such as flare during on-going treatment increases mineralization as a consequence of healing and thus increases 18F-fluoride uptake in early remission. The impact of different treatment effects and (cytotoxic versus cytostatic) tumour heterogeneity is still highly unclear and needs further evaluation. Since virtually all future studies using 18F-fluoride will use hybrid PET/CT, the combined information from both modalities might provide further insights into treatment effects. Awareness of biological uptake mechanism dynamics of 18F-fluoride at different time points and the effect of a given treatment are crucial for imaging response evaluation and require further studies.

Oncologists or urologists in charge of managing patients with prostate cancer have several choices for evaluating the treatment response in metastatic disease to the bone. CT, whole-body MRI and BS are the classical and widely available imaging modalities. Morphological assessments with CT and MRI using RECIST protocols are time-consuming. Planar BS is associated with lower sensitivity and specificity than 18F-fluoride PET/CT, but automated software for tumour burden definition is available. 18F-fluoride PET/CT has a very high sensitivity for detection of early bone metastasis in many cancers and, as shown in this study, is highly reproducible under controlled conditions. Similar to BS, the radioactive signal from 18F-fluoride is largely irrelevant for evaluating soft tissue metastases, but PET can be combined with simultaneous contrast-enhanced CT to overcome this limitation. Definition of the total skeletal tumour burden using 18F-fluoride is experimental, but the softwares used in this study are available either commercially or in the public domain. In this study, measurements of uptake and volumes from five larger index lesions had higher repeatability than total skeletal tumour burden and are potentially sufficient in clinical trials. The inclusion of small lesions in TLF reduced accuracy, which is largely associated with the resolution of both the scanner and the reconstructed image matrix. Although promising from the point of accuracy, more prospective trials are needed to validate 18F-fluoride PET/CT as a surrogate biomarker of outcome before routine application. Direct imaging of the tumour metabolic activity using 18F-FDG PET/CT is widely applicable but has a relatively low sensitivity in PCa. Malignant lipogenesis is a hallmark of aggressively growing cancer both in soft tissue and bone metastases and can be imaged using ^11^C-acetate [20, 21] or ^11^C/18F-choline PET/CT [22, 23], but these tracers are less widely available and there are few studies on accuracy and other aspects of biomarker qualification so far. Both 18F-fluoride and 18F-choline have been shown to detect more skeletal lesions than ^99m^Tc-diphosphonate bone scanning [24, 25]. 18F-fluoride is also more specific than BS [26].

## Conclusions

Quantitative 18F-fluoride uptake and biochemical tumour and specific bone remodelling markers measured at the same time point are reproducible for skeletal prostate cancer metastases. The awareness of treatment response and tumour heterogeneity is of greatest importance since 18F-fluoride quantification reflects bone remodelling and not tumour activity.
